# Measuring the structure and equation of state of polyethylene terephthalate at megabar pressures

**DOI:** 10.1038/s41598-021-91769-0

**Published:** 2021-06-18

**Authors:** J. Lütgert, J. Vorberger, N. J. Hartley, K. Voigt, M. Rödel, A. K. Schuster, A. Benuzzi-Mounaix, S. Brown, T. E. Cowan, E. Cunningham, T. Döppner, R. W. Falcone, L. B. Fletcher, E. Galtier, S. H. Glenzer, A. Laso Garcia, D. O. Gericke, P. A. Heimann, H. J. Lee, E. E. McBride, A. Pelka, I. Prencipe, A. M. Saunders, M. Schölmerich, M. Schörner, P. Sun, T. Vinci, A. Ravasio, D. Kraus

**Affiliations:** 1 Helmholtz-Zentrum Dresden-Rossendorf, Bautzner Landstrasse 400, 01328 Dresden, Germany; 2 Institute for Solid State and Materials Physics, Technische Universität Dresden, 01069 Dresden, Germany; 3 SLAC National Accelerator Laboratory, Menlo Park, CA 94025 USA; 4 LULI, CNRS, CEA, Sorbonne Université, Ecole Polytechnique - Institut Polytechnique de Paris, 91128 Palaiseau, France; 5 Institute of Nuclear and Particle Physics, Technische Universität Dresden, 01069 Dresden, Germany; 6 Lawrence Livermore National Laboratory, Livermore, CA 94550 USA; 7 Department of Physics, University of California, Berkeley, CA 94720 USA; 8 Lawrence Berkeley National Laboratory, Berkeley, CA 94720 USA; 9 CFSA, Department of Physics, University of Warwick, Coventry, CV4 7AL UK; 10 European XFEL GmbH, Holzkoppel 4, 22869 Schenefeld, Germany; 11 Institut für Physik, Albert-Einstein-Str. 23, Universität Rostock, 18059 Rostock, Germany

**Keywords:** Planetary science, Laser-produced plasmas, Theory and computation, Laboratory astrophysics

## Abstract

We present structure and equation of state (EOS) measurements of biaxially orientated polyethylene terephthalate (PET, $({\hbox {C}}_{10} {\hbox {H}}_8 {\hbox {O}}_4)_n$, also called mylar) shock-compressed to ($155 \pm 20$) GPa and ($6000 \pm 1000$) K using in situ X-ray diffraction, Doppler velocimetry, and optical pyrometry. Comparing to density functional theory molecular dynamics (DFT-MD) simulations, we find a highly correlated liquid at conditions differing from predictions by some equations of state tables, which underlines the influence of complex chemical interactions in this regime. EOS calculations from ab initio DFT-MD simulations and shock Hugoniot measurements of density, pressure and temperature confirm the discrepancy to these tables and present an experimentally benchmarked correction to the description of PET as an exemplary material to represent the mixture of light elements at planetary interior conditions.

## Introduction

The interiors of the icy giant planets of our solar system, Uranus and Neptune, are believed to be dominated by a vast mantle of so-called ‘ices’, i.e. ${\hbox {H}}_2\hbox {O}$, ${\hbox {NH}}_3$, ${\hbox {CH}}_4$ and comparable compounds^1^, below an atmosphere that mainly consists of hydrogen and helium. Inside the ice region, temperatures around 2000 K to 8000 K prevail at pressures up to several 100 GPa^1,2^. Such conditions fall within the ‘warm dense matter’ (WDM) regime, in the transition from condensed matter to the plasma state. In such environments, quantum effects have to be taken into account while temperature- and/or pressure-induced ionization or metallization also start to play a prominent role^3^. This complexity significantly impedes a precise theoretical modelling of WDM states. Nevertheless, a reliable description of the ices under intra-planetary conditions is crucial for our understanding of giant planets since models of the icy layers are highly sensitive to various physical properties like their temperature and density^4,5^.

Diamond anvil cell experiments^6–8^ and theoretical predictions^9^ suggest a polymerization of organic ices compressed to a regime relevant to the interiors of giant planets and a possible formation of diamonds. The latter could be achieved (at $P= (150 \pm 15)\, \text {GPa}$ and $T= (5000 \pm 500)\, \text {K}$) by applying laser-driven double-stage compression on polystyrene (PS, $({\text{C}}_{8}{\text{H}}_{8})_{\text{n}}$) to model a carbon-rich atmosphere^10,11^. In this paper, we discuss an experiment with a comparable setup using a simpler single-shock loading scheme and polyethylene terephthalate (PET, $({\hbox {C}}_{10}{\hbox {H}}_8 {\hbox {O}}_4)_n)$ samples to introduce oxygen to the mixture, which makes it a more realistic sample for planetary conditions, and study the oxygen’s influence on the carbon and hydrogen structure.

**Figure 1:**
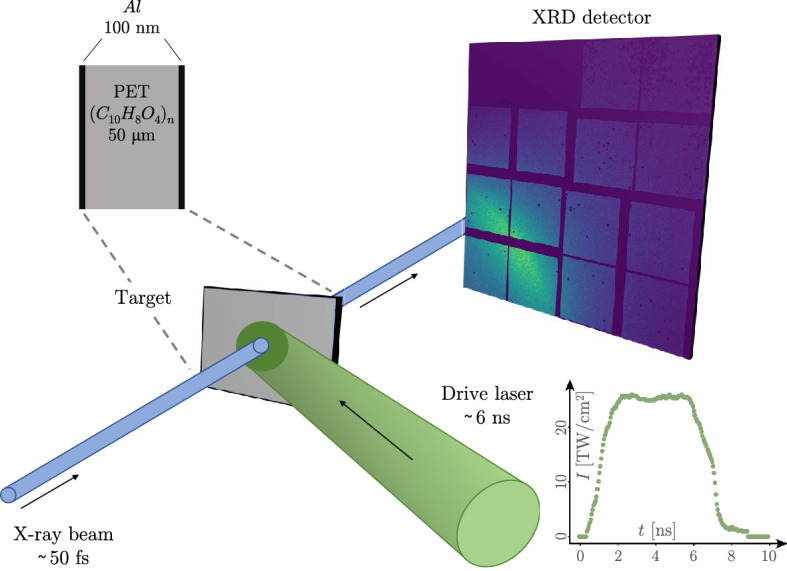
Experimental setup for the XRD measurements. A PET foil was shock-compressed using a 6 ns (FWHM) optical laser pulse. Structural changes were probed by the 8.1 keV LCLS X-ray beam via in situ XRD. The resulting diffraction image has been recorded using a single-photon-sensitive area detector.

The compressed samples were probed by in situ X-ray scattering. Comparing the resulting diffraction patterns to predictions made on the basis of density functional theory molecular dynamics (DFT-MD) simulations provides insight into the microscopic structure and an estimation for the conditions inside the target (see “X-ray diffraction measurement” section). Shock state measurements using velocity interferometer for any reflector (VISAR) and streaked optical pyrometer (SOP) diagnostics are presented in the “Shock Hugoniot measurements” section. The results are compared to Hugoniot curves obtained from different equations of state (EOS), namely SESAME 07550 Mylar^12^, PrOpacEOS 4.0.0 C5H4O2^13^ and a newly calculated EOS from ab initio DFT-MD simulations (“Comparison to different equations of state” section). This procedure allows for a straight-forward benchmark of the assumed underling EOS models.

**Figure 2:**
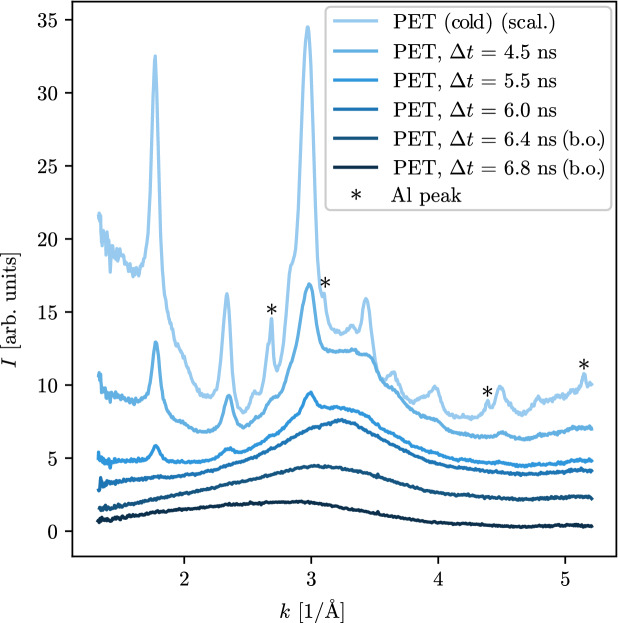
Integrated XRD data at ambient conditions and different time delays between the pump and the probe laser. The cold material shows peaks from the crystalline PET structure as well as from the aluminium coating. With the propagation of the shock front, these features recede and a signal from the amorphous liquid plastic remains, showing a wide peak centred around $k= {3.2}\, {\AA }^{-1}$. After shock breakout around $\Delta t \approx {6} \, \text {ns}$ the peak shifts to lower *k*, as it can be observed in the curves marked with “(b.o.)”. Those later delays are offset for clarity.

**Figure 3:**
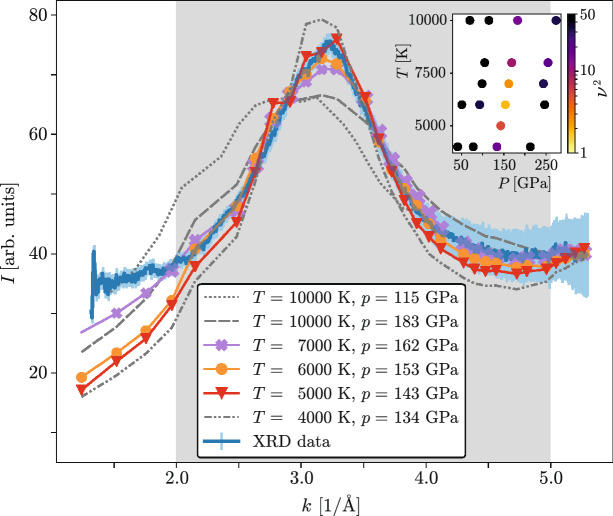
Comparison of the X-ray diffraction line-outs at the shock breakout to predictions made using density functional theory and molecular dynamics calculations. The gray region marks the *k* interval in that the simulations are fitted to the measurement. In the small plot, squared-deviations $\nu ^2$ (see Eq. (2)) are plotted for different simulation parameters. The coloured lines (representing $T= {7000} \, \text {K}$ at $P= {162} \, \text {GPa}$, $T= {6000} \, \text {K}$ at $P= {153} \, \text {GPa}$ and $T= {5000} \, \text {K}$ at $P= {143} \, \text {GPa}$) fit the experimental data best.

**Figure 4:**
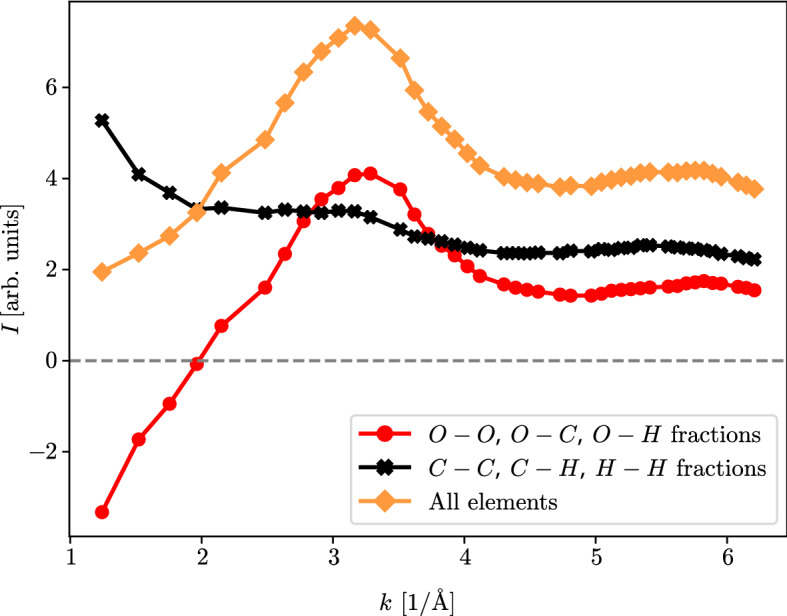
Contributions to the predicted scattering image at $T= {6000} \, \text {K}$ and $P = {153} \, \text {GPa}$ of bindings only containing C and H and those which include at least one O atom. The observed strong liquid correlations around $k= {3.2} \, {\AA }^{-1}$ seem to be caused mainly by oxygen.

## X-ray diffraction measurement

The X-ray diffraction (XRD) experiment was performed at the Matter in Extreme Conditions (MEC) end-station^14,15^ of the Linac Coherent Light Source (LCLS) at the SLAC National Accelerator Laboratory. A $50 \,\upmu \hbox {m}$ thick PET foil was irradiated with a laser pulse containing 32 J in 6 ns (full width at half maximum), focused to a spot size of $150 \, \upmu \hbox {m}$ to $200 \,\upmu \hbox {m}$. A very thin layer of the target is turned into plasma and expands rapidly, driving a shock wave into the remaining material due to the ablation pressure. To avoid transmission of the driving pulse through the target before an absorbing corona has been formed, the sample was coated with 100 nm aluminium.

We probed the compressed sample with the LCLS free electron laser X-ray beam, collecting the diffraction pattern with area detectors capable of recording single-photon events^16^. This setup (a schematic is shown in Fig. 1) is similar to previous studies on hydrocarbons^17^ but differs from experiments that found diamond formation in polystyrene^10,11^ by the choice of a single-shock loading scheme, because the Hugoniot for PET (see “Comparison to different equations-of-state” section) is expected to realize colder states at similar pressures when compared to PS.

For the analysis of the X-ray diffraction data, the signal on the detector pixels is integrated azimuthally using DIOPTAS^18^. Moreover, corrections to account for the experiment geometry are applied to the resulting 1D datasets, since the X-ray absorption inside the sample and from an attenuator in front of the detector varies with the angle of incidence.

Figure 2 shows the curves for increasing delays between the drive laser and the X-ray pulse, i.e. further transit of the shock front. The crystalline structure of the cold material results in clear peaks which recede over time as the plastic is shock-compressed and heated. By $\Delta t = {6} \, \text {ns}$ these patterns have completely vanished as the shock breaks out at the rear surface, such that there is no remaining cold material. For even longer delays, the position of the signal shifts to smaller *k* which is due to the adiabatic expansion of the material after the shock breakout.

The XRD signal recorded close to the shock breakout has been compared to DFT-MD simulations of PET under different conditions, as this is the point at which the conditions inside the target are most homogeneous. For doing so the expected X-ray scattering intensity *I* has been calculated using

1 $$\begin{aligned} I(k)&\propto \sum _{ab}\sqrt{x_ax_b} f_a(k)f_b(k) S_{ab}(k) \nonumber \\&\quad + \sum _a x_a\sum _n (1-f_{an}(k)^2){,} \end{aligned}$$

where *k* is the length of the scattering vector, $x_a$ is the atomic fraction of species *a* (with *a* being O, C or H in the case of PET), $f_a$ the atomic form factor of the whole ion or atom *a* and $f_{an}$ the contribution to $f_a$ caused by the *n*th electron. The $S_{ab}$ denote the partial structure factors.

The first line of Eq. (1) describes the elastic scattering and is obtained by assuming that the scattering occurs entirely from bound electrons, rather than screening clouds, as in the approach of Wünsch et al.^19^, which is justified by the low expected temperatures where only minor ionization should occur^20,21^. The second summand takes the inelastic scattering into account as derived by James^22^, supplemented by the summation over different species of ions or atoms. The form factors of the individual electrons in different orbitals were calculated by treating the low-*Z* atoms as hydrogen-like as it was performed by Pauling and Sherman^23^ while all other quantities were obtained from DFT-MD simulations (see “Methods” section).

Scattering intensities for densities from 2.5 to ${4.0}\, {\text {g/cm}}^{3}$ and temperatures between 4000 and 10,000 K have been evaluated (see Fig 3). As expected, a higher density shifted the position of the peak in the intensity to higher scattering vectors while an increasing temperature results in an increased peak width. To fit the artificial XRD signal to the measurement, squared deviations of the DFT-MD predictions ($C_i$) from the experimental data ($M_i$) have been calculated and weighted with the variance ($\sigma _{i}^{2}$) of the data at the given angle

2 $$\begin{aligned} \nu ^{2}=\frac{1}{N-1}\sum _{i}\frac{(M_{i}-C_{i})^{2}}{\sigma _{i}^{2}}, \end{aligned}$$

(with *N* being the number of scattering vector points) for a wave number range $\approx {2.0} \, {\AA }^{-1}<k< {5.0} \, {\AA }^{-1}$. At higher scattering vector lengths, i.e. large angles of incidence, the response function of the detector is uncertain while the underestimation of the signal for lower *k* is likely caused by rapid expansion of small fractions of the sample due to the onset of the shock release. The indicator $\nu ^2$ favours the prediction for $T= {6000} \, \text {K}$ at $P= {153} \, \text {GPa}$ and $\rho = {3.5} \, \text {g}/{\text {cm}}^{3}$ ($\nu ^2 = 1.8$) over those for identical density and $T= {7000} \, \text {K}$ at $P= {162} \, \text {GPa}$ ($\nu ^2 = 2.5$) and $T= {5000} \, \text {K}$ at $P= {143} \, \text {GPa}$ ($\nu ^2 = 5.1$) as it can be seen in the subplot of Fig. 3. By setting a threshold of acceptance we determined the uncertainty of the comparison. Hence, the specified error-intervals are a result of a parameter variation and therefore estimations.

By applying the described procedure, the conditions in the target achieved with one single shock wave have been estimated as

$$\begin{aligned} T&= (6000 \pm 1000) \, \text {K}\nonumber \\ P&= (155 \pm 20) \, \text {GPa}\nonumber \\ \rho&= (3.5 \pm 0.4) \, \text {g}/{\text {cm}}^{3}. \end{aligned}$$

Using Eq. (1), it is possible to separate the total scattering intensity into the oxygen-containing and oxygen-free components, the black and red curves, respectively, in Fig. 4. The two contributions differ substantially for $k< {4.5} \, {\AA }^{-1}$ indicating that the considerable liquid correlations around $k= {3.2} \, {\AA }^{-1}$ are caused mainly by oxygen atoms. Being negative for small *k*, the partial structure factor $S_{CO}$ lowers the intensity in this regime compared to the pure carbon and hydrogen signal.

**Figure 5:**
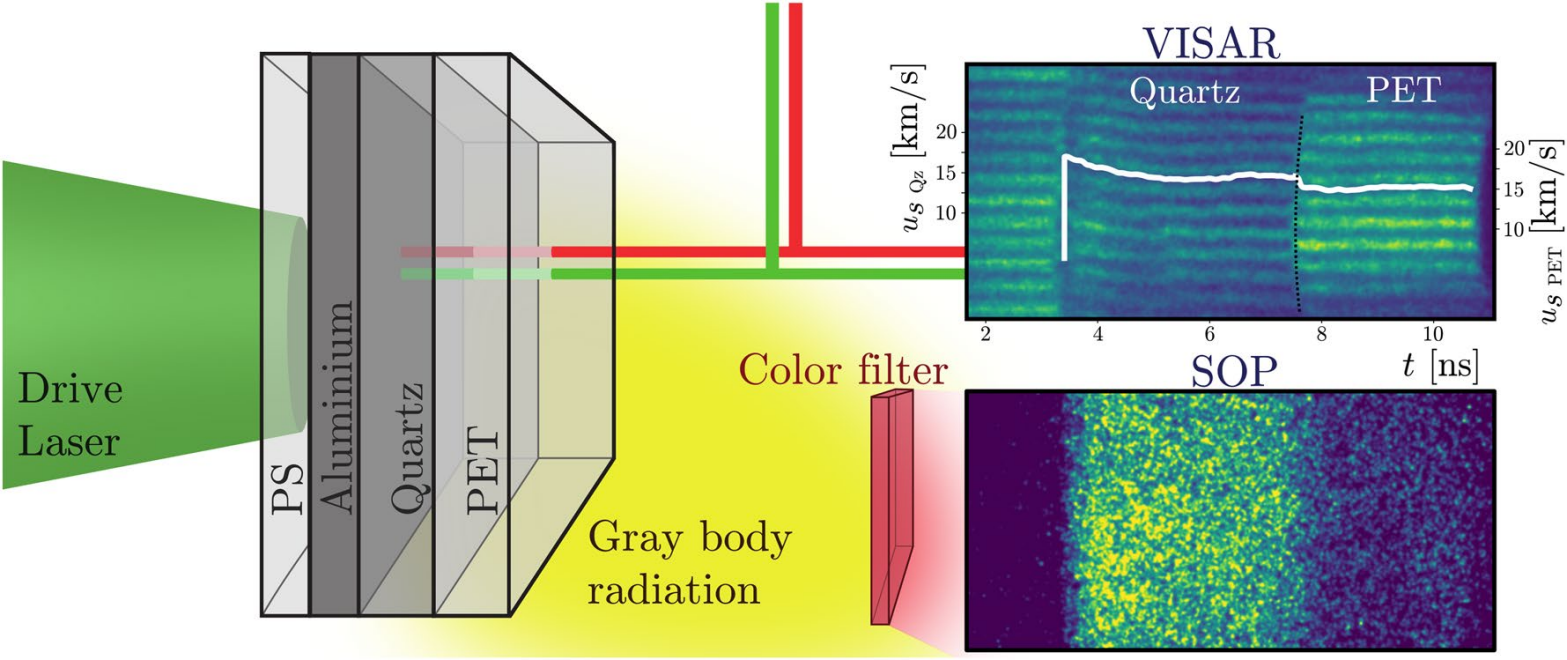
Experimental setup applied for the Hugoniot measurements. VISAR and SOP were used to diagnose the conditions inside the shock-compressed layered sample. The data shown corresponds to a drive laser energy of 400 J. Upper insert: Shock velocity extracted from VISAR fringe-shifts over time. The dashed line denotes the position where the material (and therefore the index of refraction) changes, resulting in a jump in the scale.

## Shock Hugoniot measurements

For comparing the conditions indicated by the matching of the XRD signal to the DFT-MD predictions to established Hugoniot measurement techniques, additional experiments were performed at the Laboratoire pour l’Utilisation des Lasers Intenses (LULI). Multi-component targets (layers of $\approx 10 \, \upmu \hbox {m}$ CH as an ablator, $48 \, \upmu \hbox {m}$ Al (pusher), $20 \, \upmu \hbox {m}$ to $61 \, \upmu \hbox {m}$ quartz (${\hbox {SiO}}_2$, standard) and $49 \, \upmu \hbox {m}$ PET (sample)) were dynamically shock compressed by an optical drive laser with energies from $\approx$ 236 J to 945 J. The shock velocity was measured using two velocity interferometers for any reflector (VISAR)^24^ while a calibrated streaked optical pyrometer (SOP) simultaneously provided temperature information (see Fig. 5).

To determine density and pressure in the PET, the shock velocities in quartz and PET were extracted from the VISAR fringe-shifts of the moving, reflective shock or transit times (see “Methods” section). By using a known Hugoniot for $\alpha$-quartz and an analytical release model^25,26^, pressure, density and internal energy of the PET can be calculated from impedance matching and the Rankine-Hugoniot equations.

**Figure 6:**
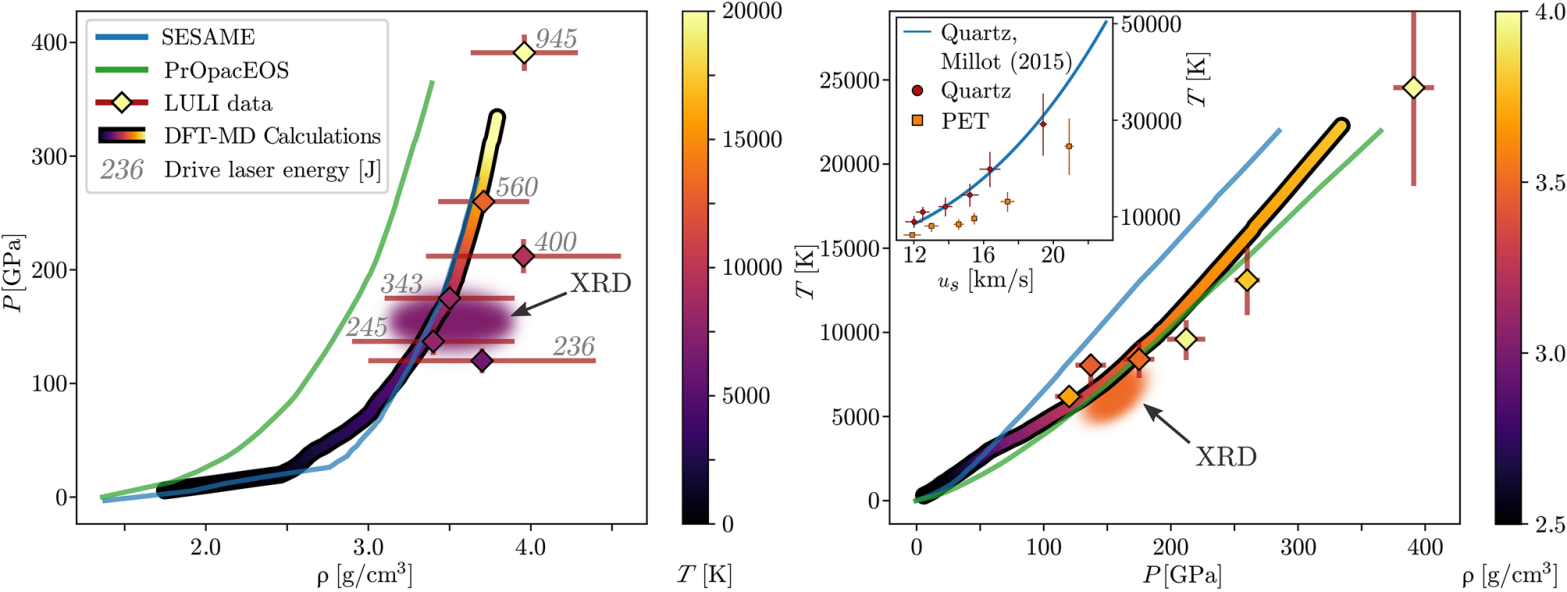
Hugoniot (left: pressure over density, right: temperature over pressure) of PET obtained from different equations of state (SESAME 07550 Mylar^12^, PrOpacEOS 4.0.0 C5H4O2^13^ and ab initio DFT-MD simulations) and VISAR and SOP measurement at the LULI facility. For the latter two, the colour indicates temperature (left) or density (right). The blurred regions indicate *T*, *P* and $\rho$ favoured by the comparison of artificial XRD images with the experiment. Numbers next to the data-points denote the energy of the laser drive. XRD, DFT-MD and LULI data seem consistent within the error margins, whereas SESAME and PrOpacEOS disagree on temperature or pressure, respectively. Right insert: Temperature vs shock velocity relation in the quartz (circles) and PET region (squares) of the samples. The blue line indicates the dependency Millot at al. obtained for quartz^27^.

The temperature of the target was determined using a SOP system to measure the emission from the shocked material while assuming the target to be a grey-body with wavelength independent reflectivity^27^. The Hugoniot curve acquired by these VISAR and SOP measurements is shown in Fig. 6.

## Comparison to different equations of state

To validate the results obtained in the “X-ray diffraction measurement” and “Shock Hugoniot measurements” sections, three Hugoniot curves for PET in the WDM regime have been calculated using different EOS. Subsequently two tables, SESAME 07550 Mylar^12^ and PrOpacEOS 4.0.0 C5H4O2^13^, will be compared to an EOS obtained from ab initio DFT-MD. Ambient conditions ($\rho _0 = {1.38} \, \text {g}/{\text {cm}}^{3}$ at room temperature and normal pressure) were set as the starting points for the Hugoniots. The resulting graphs are illustrated in Fig. 6 and allow us to compare the EOS tables with the experimental observations.

The temperatures that SESAME predicts are higher than the results of calculations based on the PrOpacEOS equation of state and DFT-MD for similar pressures. SESAME and DFT-MD agree on the pressure-density relation except in an interval between 2.5 and ${3.0} \, {\text {g/cm}}^{3}$, where pressure-induced dissociation of the molecules is expected, while PrOpacEOS predicts higher required pressures to reach the same density. The discrepancies between these graphs indicate the poor understanding of PET under WDM conditions.

The blurred regions in Fig. 6 depict the conditions that gave best agreement with the XRD pattern in the “X-ray diffraction measurement” section. The ab initio Hugoniot is compatible with this area while differences in density or temperature are found for PrOpacEOS and SESAME, respectively.

When comparing Hugoniot curves from different tables to the VISAR and SOP measurements described in the previous section, good agreement with the ab initio EOS can be found. Both SESAME and PrOpacEOS diverge considerably from both the simulated and measured data across the range of conditions probed, and provide therefore no adequate description of PET, or elemental mixtures of similar composition, at intra-planetary conditions.

To check the influence of a potential pre-heating of the sample, hydrodynamic simulations including radiation transport have been performed with the software package HELIOS from PRISM computational sciences, Inc.^13^ for SESAME and PrOpacEOS. We assumed the temperature of electrons and ions to be equal and estimated the thermal conductivity with the Spitzer-model^28^. The results of the hydro-simulations agree with the calculations from the Rankine–Hugoniot equations within the uncertainties and therefore suggest only a minor change caused by pre-heating while validating the starting conditions and interpolations used for the calculation of the Hugoniot from the EOS tables.

## Conclusions and outlook

We presented new data of polyethylene terephthalate from DFT-MD simulations and laser-shock experiments and compared it to tabulated EOS values to infer the macroscopic thermodynamic properties of PET at intra-planetary conditions. Furthermore, in-situ X-ray diffraction measurements were performed and analyzed by fitting DFT-MD based scattering image predictions to the experiment, allowing us to gain insight in both the microscopic structure and the temperature, pressure and density conditions of the sample, simultaneously.

The different methods of XRD, classical shock-state measurements and DFT-MD EOS calculations determine the state of our target consistently, complementing each other. Given this accordance, it might be worth exploring if XRD, in combination with DFT, could be utilized as a diagnostic not only for density but also temperature, e.g. for states off the Hugoniot curve.

We stress that the time scales relevant in DFT-MD simulations (ps), laser shock-experiments (ns) and planetary live-times (millions of years) differ vastly. Effects like superheating—that can occur during rapid shock compression^27,29^ but are not likely to be relevant for astrophysical objects—impede a direct analogy. Nevertheless, nano-second scale experiments allow experimental access to the WDM regime that can be used to gain valuable insight into the physics under extreme conditions.

Using the described method, we constrain the conditions inside the region probed by the X-rays to $T = (6000 \pm 1000) \, \text {K}$, $P = (155 \pm 20) \, \text {GPa}$ and $\rho = (3.5 \pm 0.4) \text {g}/{\text {cm}}^{3}$. This result disagrees with the Hugoniots of both SESAME 07550 Mylar^12^ (which predicts higher temperatures) and PrOpacEOS 4.0.0 C5H4O2^13^ (that underestimates the density). However, the conditions do fall on the Hugoniot of the EOS derived from our own DFT-MD simulations. This dataset has been compared to SOP and VISAR measurements of shock compressed PET where we found agreement within the error bars.

The regime characterized by the *T*, *P* and $\rho$ stated above is believed to be realized inside the icy planets^2,30^ and is close to the conditions where diamond formation—which might be supported by the presence of oxygen^31^—has been observed in comparable experiments with polystyrene samples^10,11^. While no conclusive statement about the crystallization of diamond from PET can be given here based on the analyzed dataset due to the notable liquid background, further experiments for obtaining insight into the possibility of this process, e.g. with thicker samples (and therefore more time for diamonds to form) or colder, weaker shocks (since the temperature we found lies only little under the diamond melting line^32^), seem promising—even though correlations of oxygen with carbon and hydrogen may impede the detection. PET seems to be a more suitable model system for the icy giants’ interior layers than PS, since it contains oxygen, but also due to the technical simplicity of generating relevant conditions using just a single shock wave.

## Methods

### XRD

We probed the sample using a $\approx {50} \, \text {fs}$ long X-ray beam with $\approx {3} \, \text {mJ}$ pulse energy focussed to a spot size of ${20} \, \upmu \text {m}$ (full width at half maximum). The photon energy amounted $E_{\text {ph}} = {8.1} \, \text {keV}\pm {0.3} \, {\%}$. The diffraction measurement was performed in an angular range from $2\theta = {20}^{\circ }\text { to } {95}^{\circ }$ (corresponding to scattering vector lengths from $k= {1.4} \, {\AA }^{-1}$ to ${6.0}\, {\AA }^{-1}$) with a ${8}\, \text {cm}\times {8} \, \text {cm}$ Cornell-Stanford Pixel Array detector^16^. Gaps in the detector and malfunctioning pixels have been masked out before the signal was integrated azimuthally. In the latter step, corrections for the polarization of the X-ray beam were applied.

### DFT-MD calculations

The DFT-MD simulations were performed using the VASP package, version 5.2^33–36^. The electronic density in the simulation box with periodic boundary conditions was represented by a plane wave expansion with a cut-off energy of $E_{\text {cut}} = {1000} \, \text {eV}$. We used the Mermin formulation of DFT to optimize the Helmholtz free energy at a given temperature^37^. The electron-ion interaction was modeled using the projector augmented wave (PAW) approach, specifically the hard PAW pseudopotentials for hydrogen (H_h, 06Feb2004), carbon (four valence electrons, C_h Feb2004), and oxygen (six valence electrons, O_h Feb2004) as provided with VASP^38,39^. The exchange-correlation potential was taken in generalized gradient approximation in Perdev-Burke-Ernzerhof parametrisation (GGA-PBE)^40^. We generally sampled the Brillouin zone of the supercell at the $\Gamma$-point only. The electronic bands where populated using a Fermi distribution at the chosen temperature. We performed simulations with two different system sizes: 32 (64) protons, 40 (80) atoms of carbon, and 16 (32) atoms of oxygen (this corresponds to four (eight) PET subunits), and their movements were calculated using the Hellman–Feynman forces derived from the electron densities of DFT under the Born–Oppenheimer approximation. The DFT-MD run covered a time span of 2 ps to 4 ps, the time step was $t = {0.2} \, \text {fs}$, and the ion temperature was controlled by a Nosé-Hoover thermostat^41^.

### VISAR

To determine the shock velocity without ambiguity, two VISAR systems with different sensitivities were included in the setup. For each, the velocity per VISAR fringe $\text {VPS}_0$ was calculated using^42,43^

3 $$\begin{aligned} \text {VPF}_0 = \frac{\lambda _L c}{4\cdot d (n - 1/n) (1+\delta )}{,} \end{aligned}$$

where $\lambda _L$ is the wavelength of the applied laser (1064 nm / 532 nm), *c* the speed of light, $n=1.4497$ or 1.4607 the wavelength dependent index of refraction of the etalon material, *d* its thickness (8.06 mm/10.04 mm) and $\delta = 0.0163$ or 0.0318 a correction for the dispersion in the etalon^43,44^. We found for the two systems $\text {VPF}_0= {12.81} \, \text {km/s/fringe}$ and $\text {VPF}_0 = {4.98} \, \text {km/s/fringe}$, respectively. To calculate the velocity per fringe in a medium, $\text {VPF}_0$ has to be divided by its index of refraction which is given as $n_{\text {Qz}} = 1.54$ and $n_{\text{PET}}=1.63$ for our targets.

For the two lowest-energy shots, where this procedure was not applicable in the quartz-region due to the low reflectivity, transit times were used to calculate the shock velocities instead. To measure this quantity, the time of shock-entry and -breakout was determined up to $\pm {0.15} \, \text {ns}$, each, and the uncertainty in the target’s thickness amounted $\pm 1 \, \upmu \hbox {m}$.

### SOP

For analyzing the SOP data, we treated the target as a grey body with wavelength-independent reflectivity *R*, measured using the VISAR system and normed to the known reflectivity in quartz^45^. With these assumptions, the spectral radiance *L* of the target is given by

4 $$\begin{aligned} L(\lambda , T) = (1-R) \frac{2 h c^2}{\lambda ^5} \frac{1}{\exp (hc /(\lambda k_b T))-1}{.} \end{aligned}$$

In this equation, *c* denotes the speed of light, *h* the Planck’s constant, $\lambda$ the radiation’s wavelength and $k_b$ Boltzmann’s constant^46,47^. Using a narrow bandwidth filter that can be described by a $\delta$ function around a given wavelength $\lambda _0$, Eq. (4) can be inverted and the temperature inside the sample calculated using

5 $$\begin{aligned} T = \frac{T_0}{\ln \left( 1 + (1-R) A / I \right) }, \end{aligned}$$

where *I* is the number of counts accumulated on the CCD chip. $T_0=\frac{hc}{\lambda _0 k_b}$ only depends on the filter used^45^ while *A* is a constant containing various machine-parameters^46^ and was obtained using the known temperature to shock velocity relation of $\alpha$-quartz^27,48^ as a reference^49^. Errors where estimated from the uncertainty of this calibration (by fitting upper and lower bounds to the reference data, resulting in relative derivations of $T_0:^{+2.5\%}_{-15\%}$ and $A:^{-10\%}_{-14\%}$ or $A:^{+39\%}_{-22\%}$ at a fixed $T_0$, depending on the filter^49^), the reflectivity measurement and the signal’s variance.

## Data Availability

The datasets generated during the experiments are available from the corresponding author on reasonable request.

## References

[1] Guillot T (2005). The interiors of giant planets: Models and outstanding questions. Annu. Rev. Earth Planet. Sci..

[2] Helled R, Anderson JD, Podolak M, Schubert G (2011). Interior models of uranus and neptune. Astrophys. J..

[3] Drake RP (2006). High-Energy-Density Physics. Shock Wave and High Pressure Phenomena.

[4] Nettelmann N (2016). Uranus evolution models with simple thermal boundary layers. Icarus.

[5] Bethkenhagen M (2017). Planetary ices and the linear mixing approximation. Astrophys. J..

[6] Benedetti LR (1999). Dissociation of CH4 at high pressures and temperatures: Diamond formation in giant planet interiors?. Science.

[7] Hirai H, Konagai K, Kawamura T, Yamamoto Y, Yagi T (2009). Polymerization and diamond formation from melting methane and their implications in ice layer of giant planets. Phys. Earth Planet. Inter..

[8] Lobanov SS (2013). Carbon precipitation from heavy hydrocarbon fluid in deep planetary interiors. Nat. Commun..

[9] Ancilotto F (1997). Dissociation of methane into hydrocarbons at extreme (planetary) pressure and temperature. Science.

[10] Kraus D (2017). Formation of diamonds in laser-compressed hydrocarbons at planetary interior conditions. Na. Astron..

[11] Kraus D (2018). High-pressure chemistry of hydrocarbons relevant to planetary interiors and inertial confinement fusion. Phys. Plasmas.

[12] Barnes, J. & Lyon, S. Sesame 7550. *Los Alamos National Laboratory Report No. LA-UR-1988-770* (1988).

[13] MacFarlane J, Golovkin I, Woodruff P (2006). HELIOS-CR—A 1-d radiation-magnetohydrodynamics code with inline atomic kinetics modeling. J. Quant. Spectrosc. Radiat. Transfer.

[14] Nagler B (2015). The matter in extreme conditions instrument at the linac coherent light source. J. Synchrotron. Radiat..

[15] Glenzer SH (2016). Matter under extreme conditions experiments at the linac coherent light source. J. Phys. B At. Mol. Opt. Phys..

[16] Hart, P. *et al.* The CSPAD megapixel x-ray camera at LCLS. In *SPIE Optical Engineering + Applications, SPIE Proc., 85040C* (eds Moeller, S. P. *et al.*) (SPIE, 2012). 10.1117/12.930924.

[17] Hartley N (2018). Liquid structure of shock-compressed hydrocarbons at megabar pressures. Phys. Rev. Lett..

[18] Prescher C, Prakapenka VB (2015). Dioptas: A program for reduction of two-dimensional X-ray diffraction data and data exploration. High Press. Res..

[19] Wünsch K, Vorberger J, Gregori G, Gericke DO (2011). X-ray scattering as a probe for warm dense mixtures and high-pressure miscibility. Europhys. Lett..

[20] Wünsch, K. *Theory of X-ray Thomson scattering in warm dense matter*. PhD thesis, University of Warwick, Warwick (2011).

[21] Kraus, D. *Characterization of phase transitions in warm dense matter with X-ray scattering: Charakterisierung von Phasenübergängen in warmer dichter Materie mit Röntgenstreuung*. PhD thesis, Technische Universität, Darmstadt (2012).

[22] James RW (1962). The Optical Principles of the Diffraction of X-rays, Vol 2 of The Crystalline State.

[23] Pauling L, Sherman J (1932). Screening Constants for Many-Electron Atoms. The Calculation and Interpretation of X-ray Term Values, and the Calculation of Atomic Scattering Factors.

[24] Barker LM, Hollenbach RE (1972). Laser interferometer for measuring high velocities of any reflecting surface. J. Appl. Phys..

[25] Knudson MD, Desjarlais MP (2013). Adiabatic release measurements in $$\alpha $$-quartz between 300 and 1200 gpa: Characterization of $$\alpha $$-quartz as a shock standard in the multimegabar regime. Phys. Rev. B.

[26] Desjarlais MP, Knudson MD, Cochrane KR (2017). Extension of the hugoniot and analytical release model of $$\alpha $$-quartz to 0.2–3 tpa. J. Appl. Phys..

[27] Millot M (2015). Shock compression of stishovite and melting of silica at planetary interior conditions. Science.

[28] Spitzer L, Härm R (1953). Transport phenomena in a completely ionized gas. Phys. Rev..

[29] White S (2020). Time-dependent effects in melting and phase change for laser-shocked iron. Phys. Rev. Res..

[30] Guillot T, Chabrier G, Gautier D, Morel P (1995). Effect of radiative transport on the evolution of Jupiter and Saturn. Astrophys. J..

[31] Chau R, Hamel S, Nellis WJ (2011). Chemical processes in the deep interior of Uranus. Nat. Commun..

[32] Wang X, Scandolo S, Car R (2005). Carbon phase diagram from ab initio molecular dynamics. Phys. Rev. Lett..

[33] Kresse G, Hafner J (1993). Ab initiomolecular dynamics for liquid metals. Phys. Rev. B.

[34] Kresse G, Hafner J (1994). Ab initiomolecular-dynamics simulation of the liquid-metal–amorphous-semiconductor transition in germanium. Phys. Rev. B.

[35] Kresse G, Furthmüller J (1996). Efficiency of ab-initio total energy calculations for metals and semiconductors using a plane-wave basis set. Comput. Mater. Sci..

[36] Kresse G, Furthmüller J (1996). Efficient iterative schemes for ab initio total-energy calculations using a plane-wave basis set. Phys. Rev. B.

[37] Mermin ND (1965). Thermal properties of the inhomogeneous electron gas. Phys. Rev..

[38] Blöchl PE (1994). Projector augmented-wave method. Phys. Rev. B.

[39] Kresse G, Joubert D (1999). From ultrasoft pseudopotentials to the projector augmented-wave method. Phys. Rev. B.

[40] Perdew JP, Burke K, Ernzerhof M (1996). Generalized gradient approximation made simple. Phys. Rev. Lett..

[41] Nosé S (1991). Constant temperature molecular dynamics methods. Prog. Theor. Phys. Suppl..

[42] Celliers PM (2004). Line-imaging velocimeter for shock diagnostics at the OMEGA laser facility. Rev. Sci. Instrum..

[43] Barker LM, Schuler KW (1974). Correction to the velocity-per-fringe relationship for the visar interferometer. J. Appl. Phys..

[44] Malitson IH (1965). Interspecimen comparison of the refractive index of fused silica. J. Opt. Soc. Am..

[45] Huser G (2015). Experimental and ab initio investigations of microscopic properties of laser-shocked Ge-doped ablator. Phys. Rev. E.

[46] Miller JE (2007). Streaked optical pyrometer system for laser-driven shock-wave experiments on OMEGA. Rev. Sci. Instrum..

[47] Bolis RM (2016). Decaying shock studies of phase transitions in MgO-SiO2 systems: Implications for the super-earths interiors: Decaying shock in MgO-SiO2 systems. Geophys. Res. Lett..

[48] Brygoo S (2015). Analysis of laser shock experiments on precompressed samples using a quartz reference and application to warm dense hydrogen and helium. J. Appl. Phys..

[49] Ravasio A (2021). Metallization of shock-compressed liquid ammonia. Phys. Rev. Lett..

